# A sustainable and secure load management model for green cloud data centres

**DOI:** 10.1038/s41598-023-27703-3

**Published:** 2023-01-10

**Authors:** Deepika Saxena, Ashutosh Kumar Singh, Chung-Nan Lee, Rajkumar Buyya

**Affiliations:** 1 Department of Computer Applications, National Institute of Technology, Kurukshetra, Haryana 136119 India; 2 Department of Computer Science, Goethe University, Frankfurt, Germany; 3 Department of Computer Science and Engineering, National Sun Yat-Sen University, Kaohsiung, 804201 Taiwan; 4 Cloud Computing and Distributed Systems (CLOUDS) Lab, School of Computing and Information Systems, University of Melbourne, Melbourne, 804201 Australia

**Keywords:** Engineering, Energy infrastructure, Energy grids and networks

## Abstract

The massive upsurge in cloud resource demand and inefficient load management stave off the sustainability of Cloud Data Centres (CDCs) resulting in high energy consumption, resource contention, excessive carbon emission, and security threats. In this context, a novel Sustainable and Secure Load Management (SaS-LM) Model is proposed to enhance the security for users with sustainability for CDCs. The model estimates and reserves the required resources viz., compute, network, and storage and dynamically adjust the load subject to maximum security and sustainability. An evolutionary optimization algorithm named Dual-Phase Black Hole Optimization (DPBHO) is proposed for optimizing a multi-layered feed-forward neural network and allowing the model to estimate resource usage and detect probable congestion. Further, DPBHO is extended to a Multi-objective DPBHO algorithm for a secure and sustainable VM allocation and management to minimize the number of active server machines, carbon emission, and resource wastage for greener CDCs. SaS-LM is implemented and evaluated using benchmark real-world Google Cluster VM traces. The proposed model is compared with state-of-the-arts which reveals its efficacy in terms of reduced carbon emission and energy consumption up to 46.9% and 43.9%, respectively with improved resource utilization up to 16.5%.

## Introduction

Nowadays, there is a strong tendency towards “digitization in everything and everything in digitization” across the globe which has increased cloud data centre (CDC) traffic exponentially. Likely, the high emission of greenhouse gases such as carbon footprints along with heat generation and shared computing-derived multi-tenant environment puts a significant question on sustainability and security of CDCs. The electrical energy consumption of CDCs would increase up to 15-fold by 2030, i.e., approximately 8 per cent of projected global demand which is estimated to account for more than 3.2 per cent of the total worldwide greenhouse gas emissions^1^. The power supply avenue has a huge impact on carbon footprint emission such as high carbon emitting source (for example, coal) dominates lower carbon sources such as renewable energy (for example, wind, sun) in carbon footprint production^2,3^. Therefore, by establishing the proactive sustainability and efficiency measures at inception, and leveraging the latest technology CDCs have to explore using renewable energy such as wind, hydro or solar to power data centres and optimising technology to improve its efficiency and operating temperature while reducing carbon emission^4^. Several factors contribute to the energy and carbon efficiency of CDCs^5,6^ which must be considered during physical resource distribution and management based on environmental criteria. These factors include higher average utilization of physical server machines via virtualization; green power supply to the servers employing renewable sources of energy for reduced carbon emission; improved power usage efficiency (PUE) of the servers to save potential carbon emission; energy-efficient utilization of server machines while delivering cloud services to the end-users^7^. Among these, the most significant factor is efficient management of load while distributing physical resources which directly affects the server utilization, PUE and security of CDCs^7,8^. Nevertheless, while accomplishing a green cloud computing environment, an essential requirement of the cloud user i.e., security of application data during processing as well as storage should not be neglected^9^. Co-residency of multiple users sharing the same server machine maximizes the probability of security threats such as data hampering, leakage of sensitive information etc^10^. This gives a motivation to develop an effective solution for secure and sustainable cloud resource distribution and load management.

The major challenge entangled with developing such a solution is the trade-off about the contradictory objectives during load management. Undeniably, the cloud service provider aspires to maximize the revenues by distributing maximum workload on the minimum number of active servers to exhilarate energy efficiency and reduce power consumption costs while ignoring the security aspects during load execution. Such a distribution of resources allows multiple users to share the common physical machines and accelerates the probability of security breaches on VMs executing the workload of different users. Contrary to this, energy efficiency of the cloud environment descends and carbon footprint emission rises if the CSP minimizes sharing of the physical servers to strengthen the security of users’ workload.

In view of the aforementioned context, this article proposes a novel **S**ecure and **S**ustainable **L**oad **M**anagement (**SaS-LM**) Model to minimize the security threats, power consumption, and carbon emission and maximize server resource utilization and PUE. This model analyses cloud workload in anticipation while addressing different resource utilization on virtual machines and manages the entire load while considering multiple factors related to security and sustainability. It employs a Multi-layered Feed Forward Neural Network (MFNN) as a workload analyser which is optimized by a newly developed Dual-Phase BlackHole Optimization (DPBHO) algorithm. Further, a secure and sustainable VM placement (VMP) is presented for optimized allocation of physical resource among VMs to serve the perspectives of both cloud user and service providers while procuring sustainability of CDCs. For the cloud users, it ingrains the secure placement of VMs by minimizing the probability of security breaches and reduces the operational cost of CDC for service provider by maximizing server resource utilization and minimizing power consumption. Also, the sustainability of the cloud environment is enhanced by improving power usage effectiveness and minimizing carbon footprint intensity.

The key contributions of the proposed work are fivefold:

- MFNN-based cloud workload resource usage analyser is developed to forecast resource usage in real-time with enhanced accuracy which triggers load shifting to alleviate the effect of over/under-load on the server before its actual occurrence and improve performance of CDC.
- A novel DPBHO algorithm is proposed for optimization of MFNN during cloud resource usage estimation. It is further extended to a multi-objective DPBHO (i.e., M-DPBHO) for placement of VMs subject to multiple constraints and objectives.
- Secure and sustainable VMP is proposed to procure sustainability, energy consumption and security of CDC, simultaneously serving the perspectives of both service provider as well as end-user.
- It facilitates the secure execution of user applications by minimizing the resource sharing among users of common physical server machines in real-time.
- The experimental simulation and evaluation of the proposed model by using a real benchmark dataset reveal that the proposed work outperforms state-of-the-art approaches in terms of various performance metrics.

*The rest of the paper is organized as follows*: Section “Results” discusses experimental set-up and results of workload prediction, resource utilization, power consumption, sustainability, security, and trade-off among the obtained results. The proposed method is discussed in Section “Method” includes Dual-phase Black-Hole Optimization, cloud workload usage analysis, secure and sustainable VM placement, and VM management and SaS-LM operational summary. The background and related discussion is given in Section “Background and discussion”. Finally, Section “Conclusion and future work” entails conclusive remarks and future scope of the proposed work.

## Results

The simulation experiments are executed on a server machine assembled with two Intel^®^ Xeon^®^ Silver 4114 CPUs with 40 core processors and a 2.20 GHz clock speed. The server machine is deployed with 64-bit Ubuntu 16.04 LTS, having main memory of 128 GB. The data centre environment included three different types of servers and four types of VMs configuration shown in Tables 1 and 2. The resource features like power consumption ($PW_{max}, PW_{min}$), MIPS, RAM, and memory are taken from real server IBM^11^ configurations where $S_1$ is ‘ProLiantM110G5XEON3075’, $S_2$ is ‘IBMX3250Xeonx3480’ and $S_3$ is ‘IBM3550Xeonx5675’. The VMs configuration is inspired by the VM instances of the Amazon website^12^. Table 3 shows the experimental set-up parameters and their values.

**Table 1:** Server configuration.

Server	PE	MIPS	RAM (GB)	$PW_{max}$	$PW_{min}$/$PW_{idle}$
$S_1$	2	2660	4	135	93.7
$S_2$	4	3067	8	113	42.3
$S_3$	12	3067	16	222	58.4

**Table 2:** VM configuration.

VM type	PE	MIPS	RAM (GB)
$v_{small}$	1	500	0.5
$v_{medium}$	2	1000	1
$v_{large}$	3	1500	2
$v_{Xlarge}$	4	2000	3

**Table 3:** Experimental set-up parameters and their values.

Parameter	Value
Number of VMs	200-1000
Number of PMs	100-500
Number of users	60-300
Total time-period	400 mins
Periodic time-interval {$t_1$, $t_2$}	5 mins
Number of failure-prone VMs ($V^{fp}$)	20%, 50%, 80%
Number of malicious users ($U^{Mal}$)	20%, 50%, 80%
Number of VMs associated to a user	Random within range [1–8]
Temperature for cooling rackspace ($T_{in}$)	20 °C

Google Cluster Dataset (GCD) is utilized for performance estimation of SaS-LM and comparative approaches which contains resources CPU, memory, disk I/O request and resource usage information of 672,300 jobs executed on 12,500 servers for the period of 29 days^13^. The CPU and memory utilization percentage of VMs are obtained from the given CPU and memory usage percentage for each task in every five minutes over period of twenty-four hours.

Table 4 reports the performance metrics: *MAE* ($\varpi ^{MAE}$), *MSE* ($\varpi ^{MSE}$), PUE, carbon footprint rate (*CFR*), resource contention rate (*RCR*), probability of co-residency threats ($\Xi$), power consumption (*PW*), resource utilization (*RU*), the number of VM migrations (*Mig*#), and SLA violation ($SLA^V$) achieved for GCD workloads for varying sizes of the data centre (200–1000 VMs) over 400 minutes.

**Table 4:** Performance metrics for GCD workloads.

VM#	*T* (*min*.)	$\varpi ^{MAE}$	$\varpi ^{MSE}$	*PUE*	*CFR* (Kg/KWH)	*RCR* (%)	$\Xi$ (%)	*PW* (KW)	*RU* (%)	*Mig*#	$SLA^V$ (%)
200	100	0.0297	0.0023	1.34	16.51	2.17	18.12	7.86	80.1	91	2.25
	200	0.0168	0.0063	1.26	18.86	3.88	18.12	8.98	79.3	80	2.15
	300	0.0147	0.0006	1.34	20.77	1.92	18.12	9.89	79.7	77	1.85
	400	0.0126	0.0033	1.26	17.18	2.66	18.12	8.18	79.9	82	1.55
400	100	0.0413	0.0076	1.26	22.43	4.34	13.61	10.68	79.1	207	1.90
	200	0.0576	0.0009	1.24	21.40	5.31	13.62	10.19	78.6	198	2.05
	300	0.0781	0.0022	1.23	25.41	4.53	13.62	12.10	78.6	172	2.22
	400	0.0158	0.0017	1.18	23.26	6.55	13.60	11.08	78.9	176	1.95
600	100	0.0132	0.0011	1.18	37.92	1.92	19.15	14.05	79.5	274	2.81
	200	0.0199	0.0090	1.16	30.56	2.42	19.15	14.55	79.1	280	2.61
	300	0.0199	0.0031	1.08	28.81	1.25	19.15	13.81	79.2	268	1.95
	400	0.0187	0.0086	1.09	36.62	1.62	19.15	14.43	79.7	290	2.23
800	100	0.093	0.0062	1.25	52.44	3.71	21.67	24.97	78.8	360	2.125
	200	0.0116	0.0002	1.21	41.85	2.80	21.67	19.93	78.6	335	2.05
	300	0.0205	0.0042	1.22	48.49	2.64	21.67	23.09	78.6	335	2.125
	400	0.0108	0.0016	1.21	51.76	1.89	21.67	24.65	78.7	312	0.81
1000	100	0.0693	0.0033	1.12	60.61	1.61	17.71	28.86	78.5	507	3.53
	200	0.0771	0.0070	1.11	58.76	1.97	17.70	27.98	79.6	449	3.26
	300	0.0614	0.0018	1.14	54.01	3.70	17.71	25.76	79.7	453	2.08
	400	0.0388	0.0043	1.13	57.54	2.73	17.71	27.40	79.7	448	1.96

$\varpi ^{MAE}$: MAE average, $\varpi ^{MSE}$: MSE average, *PUE*: power usage efficiency, *CFR*: carbon foot-print rate, *RCR*: resource contention rate, $\Xi$: probability of co-residency attack, *PW*: power consumption, *RU*: resource utilization, *Mig*#: number of VM migrations, $SLA^V$: SLA violation.

**Figure 1:**
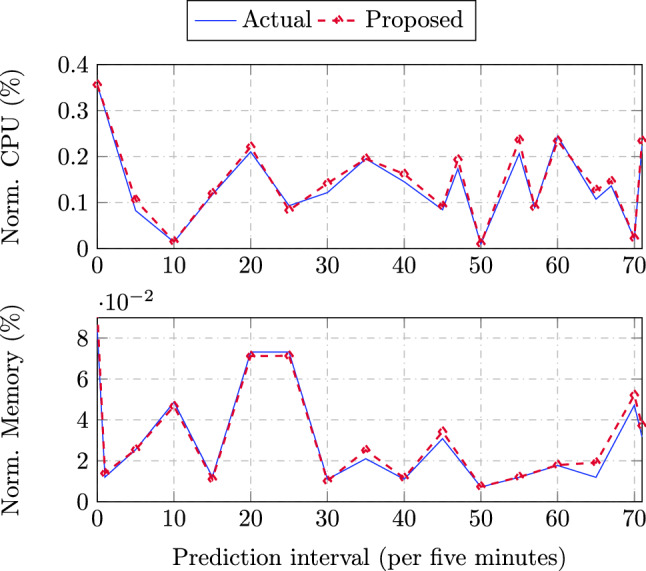
CPU and memory prediction accuracy.

The accuracy of forthcoming workload estimation using the proposed DPBHO optimized MFNN prediction unit governs the performance of the SaS-LM model. The average of failure prediction errors $\varpi ^{MAE}$ and $\varpi ^{MSE}$ vary from 0.093 to 0.0126 and 0.0090 to 0.0006, respectively. The value of *PUE* is observed in the range 1 and 1.4 which signifies the sustainable efficiency of SaS-LM. The values of *CFR* vary in line with the power consumption (*PW*) which increase with the increasing size of the data centre. The value of *PW* depends on the workload execution and the number of active servers at a specific instance. Hence, *PW* changes non-uniformly over the observed period. The *RCR* varies non-uniformly for the various sizes of data centre. The resource utilization is obtained closer to 80% which is independent of the size of the data centre. The number of VM migrations and SLA violations vary according to the variation of the workload i.e., the number of over-/under-loads experienced over a continuous period. Figure 1 plots the actual versus predicted normalized values of CPU and memory usage achieved via multiple resource prediction using MFNN, wherein the predicted values lie closer to or overlaps the actual values revealing its efficacy in terms of prediction accuracy.

The proposed work is compared for different performance metrics with various state-of-the-art approaches including Slack and Battery Aware placement (SBA)^14^, Static THReshold with Multiple Usage Prediction (THR-P) and Dynamic threshold based on Local Regression with Multiple Usage Prediction (LR-P)^15^, Previously Co-located User First (PCUF)^16^, Prediction based Energy-aware Fault-tolerant Scheduling (PEFS)^17^, Online VM Prediction based Multi-objective Load Balancing (OP-MLB)^18^, Boruta-forest optimization based Multi-objective Job Scheduling (BM-JS)^4^, VM placement with Online Multiple resources-based Feed-forward Neural Network (OM-FNN)^19^, Secure and Multi-objective VM placement (SVMP)^20^, and Wiener filter Prediction with Safety Margin (WP-SM) based VM allocation^21^. The concise description of all these approaches is provided in the discussion of Background and Table 5 presents a comparison of key performance indicators of proposed framework versus comparative approaches.

**Table 5:** Key performance indicators analysis.

KPI	^18^	^21^	^4^	^14^	^19^	^22^	^17^	^20^	^16^	SaS-LM
$\varpi ^{MAE}$	$\times$	$\times$	$\times$	$\times$	$\times$	$\times$	$\times$	$\times$	$\times$	$\checkmark$
$\varpi ^{MSE}$	$\checkmark$	$\times$	$\times$	$\times$	$\checkmark$	$\times$	$\times$	$\times$	$\times$	$\checkmark$
$Acu^{Pr}$	$\checkmark$	$\checkmark$	$\times$	$\times$	$\checkmark$	$\checkmark$	$\times$	$\times$	$\times$	$\checkmark$
*PUE*	$\times$	$\times$	$\checkmark$	$\times$	$\times$	$\times$	$\times$	$\times$	$\times$	$\checkmark$
*RCR*	$\checkmark$	$\checkmark$	$\times$	$\times$	$\times$	$\times$	$\times$	$\times$	$\times$	$\checkmark$
*CFR*	$\times$	$\times$	$\checkmark$	$\times$	$\times$	$\times$	$\times$	$\times$	$\times$	$\checkmark$
$\Xi$	$\times$	$\times$	$\times$	$\times$	$\times$	$\times$	$\times$	$\checkmark$	$\checkmark$	$\checkmark$
*RU*	$\checkmark$	$\checkmark$	$\checkmark$	$\checkmark$	$\checkmark$	$\times$	$\checkmark$	$\checkmark$	$\checkmark$	$\checkmark$
*PW*	$\checkmark$	$\checkmark$	$\checkmark$	$\checkmark$	$\checkmark$	$\checkmark$	$\checkmark$	$\checkmark$	$\times$	$\checkmark$
$A_{servers}$	$\checkmark$	$\checkmark$	$\checkmark$	$\checkmark$	$\checkmark$	$\checkmark$	$\times$	$\checkmark$	$\times$	$\checkmark$

$\varpi ^{MAE}$: mean absolute prediction error, $\varpi ^{MSE}$: mean squared error, $A_{servers}$: Active servers, $Acu^{Pr}$: Prediction accuracy, *PUE*: power usage effectiveness, *RCR*: resource contention rate, *CFR*: carbon foot-print rate, $\Xi$: probability of security threat, *RU*: Resource utilization, *PW*: Power consumption.

### Workload prediction

**Figure 2:**
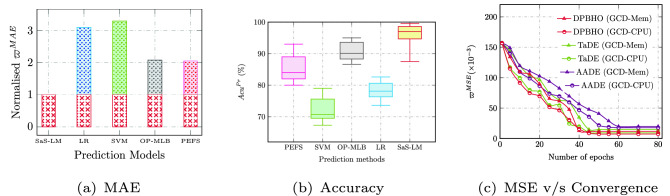
Prediction analysis.

The performance of the DPBHO optimized MFNN predictor is shown in Fig. 2, wherein Fig. 2a compares the prediction error $\varpi ^{MAE}$ normalized concerning MAE obtained for SaS-LM model. Accordingly, the box-plot based comparison of resource prediction accuracy is observed in Fig. 2b which reveals a prediction accuracy ($Acu^{Pr}$ %) trend: SaS-LM $\ge$ OP-MLB $\ge$ PEFS $\ge$ tri-adaptive differential evolution based neural network (TaDE-NN) $\ge$ auto-adaptive differential evolution based neural network (AADE-NN). The convergence capability of the proposed DPBHO algorithm while optimizing neural network based predictor, is compared with that of AADE^18^ and TaDE^19^ algorithms in Fig. 2c. DPBHO optimizes faster than AADE and TaDE while reducing prediction error ($\varpi ^{MSE}$) up to 33.3% and 19.8% over AADE and TaDE, respectively.

### Resource utilization

**Figure 3:**
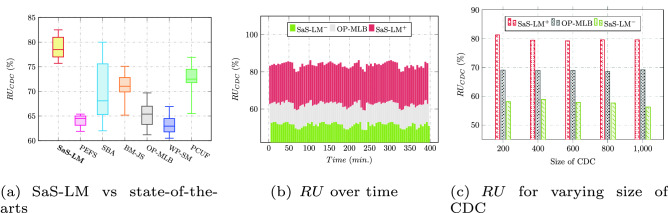
Resource utilization.

Figure 3a compares the resource utilization ($RU_{CDC}$(%)) of SaS-LM model with that of state-of-the-art approaches: PCUF^16^, PEFS^17^, SBA^14^, BM-JS^4^, OP-MLB^18^, and WP-SM^21^. All the quartiles viz., lower, upper, and median of the proposed model are higher than the respective values of quartiles of the compared approaches which indicates effectiveness of the proposed model in enhancing the $RU_{CDC}$(%). Specifically, it improves the average utilization of resources up to 14.67%, 11.4%, 7.3%, 13.2%, 16.5%, and 5.1% over PEFS, SBA, BM-JS, OP-MLB, WP-SM, and PCUF, respectively. The periodic values of $RU_{CDC}$(%) observed during time-period of 400 minutes for CDC of size 600 VMs is shown in Fig. 3b. The $RU_{CDC}$(%) obtained for varying size of CDC for SaS-LM, OP-MLB, and without SaS-LM (SaS-LM$^-$) is reported in Fig. 3c which depicts $RU_{CDC}$(%) is independent of the size of CDC.

### Power consumption

**Figure 4:**
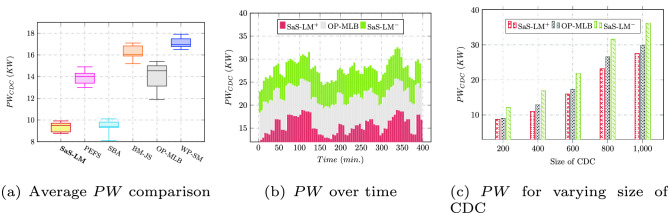
Power consumption.

The comparison of consumption of power ($PW_{CDC}$(KW)) is presented in Fig. 4a for CDC of size 200 VMs via box-plots, where SaS-LM reduced $PW_{CDC}$ up to 32.1%, 1%, 40.8%, 34.6%, and 43.9%, respectively over PEFS, SBA, BM-JS, OP-MLB, and WP-SM, respectively. Figure 4b compares the periodic values of consumption of power noticed for SaS-LM, OP-MLB, and without SaS-LM (SaS-LM$^-$) over the period of 400 minutes. The $PW_{CDC}$ obtained for varying size of CDC for the compared approaches (SaS-LM$^-$) is reported in Fig. 4c that depicts $PW_{CDC}$ rises with the size of CDC.

### Sustainability

**Figure 5:**
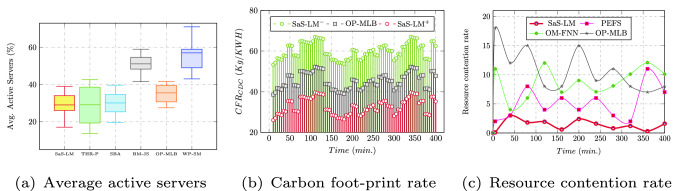
Sustainability metrics.

Figure 5a compares the average percent of active servers of SaS-LM with the related approaches. The number of active servers for SaS-LM are observed in the range [18–40%] which are reduced by 8.45%, 1.5%, 33.8%, 6.25%, and 43.5% against THR-P, SBA, BM-JS, OP-MLB, and WP-SM, respectively. The generation of carbon foot-print ($CFR_{CDC}$ (Kg/KWH)) is observed inline with the consumption of power as depicted in Fig. 5b, where the $CFR_{CDC}$ is compared over a periodic interval of 400 mins for CDC of size 600 VMs. SaS-LM has reduced the $CFR_{CDC}$ up to 21.2% and 46.9% against OP-MLB and SaS-LM$^-$, respectively. Further, the rate of resource contention realized for the related approaches is compared in Fig. 5c. The rate of failure of resources is below 4% for SaS-LM during all the experimental cases. Also, the rate of contention of physical resources is reduced up to 95.4%, 92.8%, and 89.4% over PEFS, OM-FNN, and OP-MLB, respectively.

The reason behind this performance improvement is the accurate estimation of required resources due to employment of proposed DPBHO for optimization of MFNN to allow intuitive pattern learning. Furthermore, to be acknowledged that the proposed multi-objective DPBHO has selected the most admissible VM placement strategy to enhance the resource utilization and minimize the power consumption by reducing the number of active servers while maintaining the resource availability constraints.

### Security

**Figure 6:**
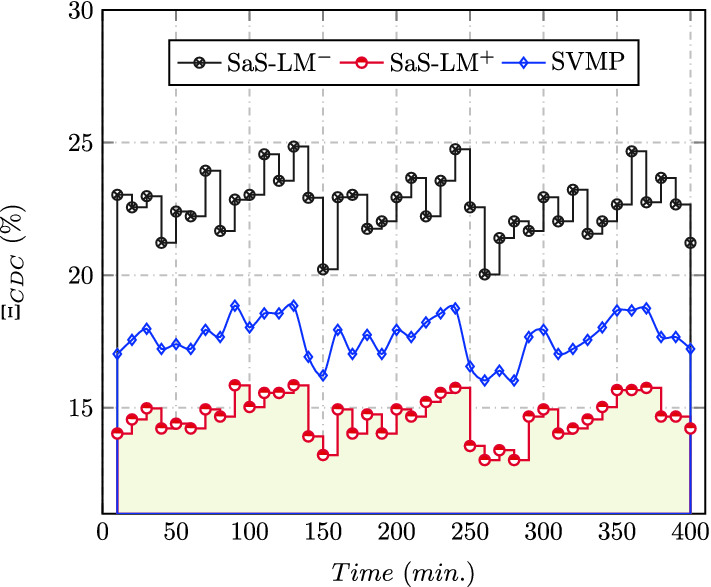
Security.

Figure 6 noted the comparison for average security breaches ($\Xi$ (%)) among SaS-LM and the relevant state-of-the-art approaches over 400 mins. The resulted values for SaS-LM are the least ($\le$ 15.1%) among all the compared approaches. The security breaches are reduced up to 17.4% and 36.4% over SVMP^20^ and SaS-LM$^-$, respectively for CDC of size 600 VMs. Table 6 compares the average co-residency resistance (%) of SaS-LM with SVMP^20^, PCUF^16^, and SaS-LM$^-$ for 600 VMs with malicious users in the range (1–10%).

**Table 6:** Comparison of average co-residency resistance (%).

VMs	$U^{Mal}(\%)$	SVMP^20^	PCUF^16^	SaS-LM$^-$	SaS-LM
600	1–10%	94–82%	95–75%	80–62%	97–84%

### Statistical analysis

The achieved results for DPBHO and M-DPBHO algorithms are validated via statistical analysis on STAC^23^ web platform using the Friedman test followed by Finner post hoc analysis in Tables 7 and 8, respectively. The Friedman test considers a null hypothesis ($H_0$) by assuming that there is no significant difference in the results of comparative approaches and assigns ranks to them based on the resultant values. The Finner post hoc test estimates the pairwise performance of the considered algorithms. The tests are conducted by using DPBHO algorithm as a control method with a significance level of 0.05 for both DPBHO and M-DPBHO algorithms. As depicted in Table 7, the Finner test accepts the $H_0$ for DNN^17^, AADE^18^, and LR algorithms which indicates the absence of a significant difference in the obtained results. However, it is rejected for comparison with SVM algorithm specifying the presence of significant difference among the observed results. Similarly, M-DPBHO obtains the best rank among all the comparative approaches as shown in Table 8. The hypothesis $H_0$ is accepted for all the comparisons revealing the absence of significant difference among all the resultant values.

**Table 7:** Statistical analysis: DPBHO v/s comparative approaches.

Friedman test			
Algorithm	Rank		
DPBHO	1.000		
DNN^17^	2.000		
AADE^18^	3.000		
LR	4.000		
SVM	5.000		

Finner Post-hoc analysis (Using DPBHO as control method)			
Comparison	Statistics	Adjusted p-value	Result
DPBHO v/s DNN	0.77460	0.43858	$H_0$ is accepted
DPBHO v/s AADE	1.80739	0.09314	$H_0$ is accepted
DPBHO v/s LR	2.06559	0.07622	$H_0$ is accepted
DPBHO v/s SVM	3.09839	0.00776	$H_0$ is rejected

**Table 8:** Statistical analysis report for M-DPBHO v/s state-of-the-art approaches.

**Friedman test**			
Algorithm	Rank		
M-DPBHO	1.000		
SBA^14^	2.000		
PEFS^17^	3.000		
OP-MLB^18^	4.000		
BM-JS^4^	5.000		
WP-SM^21^	6.000		

Finner Post-hoc analysis (Using M-DPBHO as control method)			
Comparison	Statistics	Adjusted p-value	Result
M-DPBHO v/s SBA	0.37796	0.70546	$H_0$ is accepted
M-DPBHO v/s PEFS	0.75593	0.52602	$H_0$ is accepted
M-DPBHO v/s OP-MLB	1.13389	0.39027	$H_0$ is accepted
M-DPBHO v/s BM-JS	1.51186	0.29517	$H_0$ is accepted
M-DPBHO v/s WP-SM	1.88982	0.26133	$H_0$ is accepted

### Trade-offs

There are noticeable trade-offs among resource utilization, power consumption, sustainability, and security during load management. The consolidation of VMs on a minimum number of physical machines reduces the consumption of power and wastage of resources which leads to reduced carbon footprint emissions. However, the probability of security threats increases with high virtualization and sharing of physical resources because of the multi-tenant environment. Furthermore, to enable smaller power consumption, the entire load must be allocated on the minimum number of servers which may incur resource contention among VMs and degrades security and overall performance. Hence, the sustainability improves at the cost of security at the resource management level unveiling a high contradiction between the two objectives.

## Method

**Figure 7:**
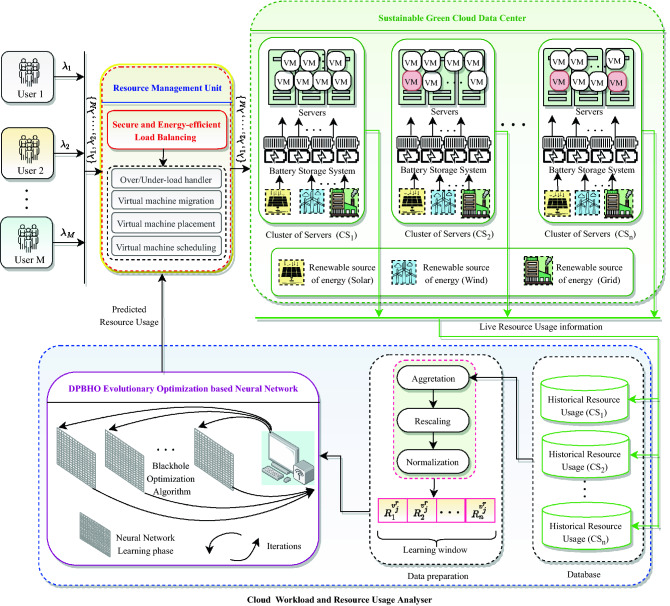
System architecture of the proposed model.

A *Sustainable CDC* infrastructure is organized utilizing *P* servers {$S_1$, $S_2$, …, $S_P$} located within *n* clusters {$CS_1$, $CS_2$, …, $CS_n$}, powered by *Renewable Source of Energy* (RSE) and grid via battery energy storage system as illustrated in Fig. 7. The electric power produced by multiple RSE such as solar panels, wind energy, and power grid charge battery storage including Uninterruptible Power Supply (UPS) which is discharged to provide required power supply and backup to clusters of servers {$CS_1$, $CS_2$, ..., $CS_n$}. Consider *M* users {$U_1$, $U_2$, …, $U_M$} submit job requests {$\lambda _1$, $\lambda _2$, …, $\lambda _M$} for execution on their purchased VMs {$V_1$, $V_2$, …, $V_Q$}$: M<Q$, where *Q* is a total number of available VMs and one job may execute on multiple VMs.

A *Resource Management Unit* (RMU) is set up to receive and distribute these requests among VMs deployed on servers {$S_1$, $S_2$, …, $S_P$}. RMU is employed to acquiesce secure and energy-efficient resource distribution based load balancing for sustainability and security augmentation within CDC. Further, it controls all the privileges of physical resource management such as handling of over-/under-loading of servers, VM placement, VM migration, scheduling etc. RMU is obliged for two-phase scheduling including (*i*) distribution of job requests {$\lambda _1$, $\lambda _2$, …, $\lambda _M$} among VMs and (*ii*) placement of VMs {$V_1$, $V_2$, …, $V_Q$} on servers. Accordingly, it assigns job requests {$\lambda _1$, $\lambda _2$, ..., $\lambda _M$} among VMs corresponding to the user specified resource (viz., CPU, memory, bandwidth) capacity. Further, it appoints a *multi-objective load balancing optimization* for allocation of users’ VMs {$V_1$, $V_2$, ..., $V_Q$} to available physical servers {$S_1$, $S_2$, ..., $S_P$} subject to security and energy-efficiency.

A *Cloud Workload and Resource Usage Analyser* (CW-RUA) is employed to estimate the workload and physical resource usage proactively and assist RMU by providing useful knowledge of resource provisioning in anticipation. CW-RUA captures the historical and live traces of resource utilization by VMs {$V_1$, $V_2$, ..., $V_Q$} hosted on different servers {$S_1$, $S_2$, ..., $S_P$} within clusters {$CS_1$, $CS_2$, ..., $CS_n$}. The workload and resource usage analysis is performed in two steps: (*i*) *Data preparation* and (*ii*) *Predictor optimization* which are executed periodically. Data is prepared in the form of a vector of learning window using three consecutive steps including *aggregation* of resource usage traces, *rescaling* of aggregated values, followed by *normalization*. The learning window vector is passed to a neural network-based predictor which is trained/optimized with the help of a novel DPBHO evolutionary optimization algorithm. The detailed description of DPBHO, CW-RUA and *Secure and Sustainable VMP* (SS-VMP) is elucidated in Sections “Dual-phase black-hole optimization”, “Cloud workload resource usage analysis” and “Secure and sustainable VM placement”, respectively.

### Dual-phase black-hole optimization

**Figure 8:**
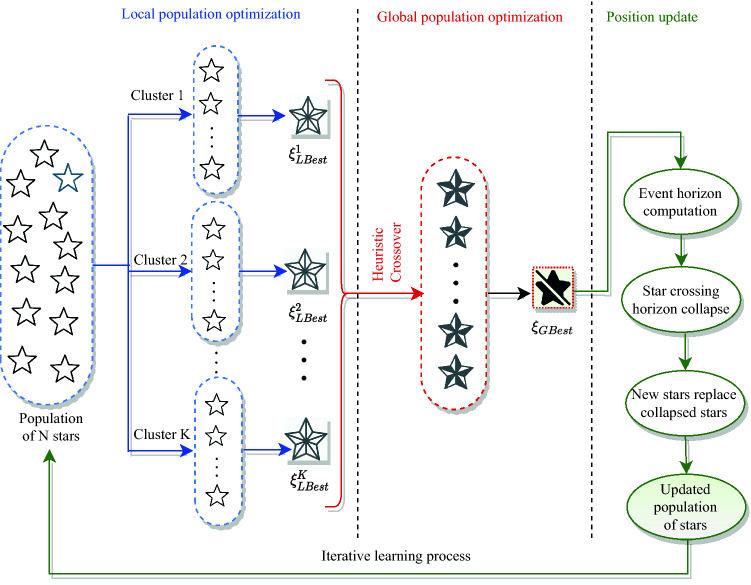
DPBHO design.

A two-phase population-based optimization algorithm named *Dual Phase Black-Hole Optimization * (DPBHO) is proposed, wherein each phase, the candidate solutions are considered as stars while a star with the best fitness value is observed as a black-hole. Figure 8 portrays the DPBHO design which incorporates three consecutive steps: (i) *Local population optimization*, (ii) *Global population optimization*, and (iii) *Position Update*.

#### Local population optimization

In this phase, the stars i.e., random solutions {$\xi _1$, $\xi _2$, …, $\xi _\mathbb {N}$}$\in \mathbb {E}$ are organized into *K* clusters or sub-populations, each of size $\mathbb {N}/K$. All the members of each cluster ($\xi _i^k: i \in [1, \mathbb {N}/K], k \in [1, K]$) are evaluated over training data using fitness value ($f_i^k$) obtained by computing Eq. (1), where $F(\xi _i^k)$ is a fitness evaluation function. The best solution of each $k^{th}$ cluster is considered as its local blackhole ($\xi ^{k}_{Lbest}$) such that $\xi ^{k}_{Lbest} =$ Best({$\xi _1$, $\xi _2$, …, $\xi _{\mathbb {N}/K}$}).

1 $$\begin{aligned} f_i^k = F(\xi _i^k) \quad \forall i \in [1, \mathbb {N}/K], k \in [1, K] \end{aligned}$$

#### Global population optimization

In the global optimization phase, all the local blackholes consitute the second phase population {$\xi ^{1}_{Lbest}$, $\xi ^{2}_{Lbest}$, …, $\xi ^{K}_{Lbest}$}, wherein *heuristic crossover* is performed to raise diversity of the second phase population by producing new individuals with a superior breed. In the course of heuristic crossover, stars act as chromosomes, where two parent chromosomes are randomly chosen and their fitness values are compared to find out the parent with better fitness value. Afterward, a new offspring is produced with the combination of two parent chromosomes using Eq. (2) which is closer to the parent having better fitness value^24^. This additional step brings significant diversity in the search space by adding new and better individuals in the second phase population. Let $\xi ^{k}_{Lbest}$ and $\xi ^{j}_{Lbest}$ be two parent chromosomes, wherein $\xi ^{k}_{Lbest}$ is considered as a parent chromosome with better fitness value. Thereafter, the offspring $\xi _{Off}$ is generated as follows:

2 $$\begin{aligned} \xi _{Off} = Cr_i(\xi ^{k}_{Lbest_i}-\xi ^{j}_{Lbest_i}) + \xi ^{k}_{Lbest_i} \quad i \in [1, L] \end{aligned}$$

where, $Cr_i$ is a randomly generated crossover rate in the range [0, 1] for $i^{th}$ gene such that *i* =$\{1,2, \ldots , L\}$, $\xi _{Off}$ is new offspring, $\xi ^{k}_{Lbest_i}$ and $\xi ^{j}_{Lbest_i}$ are $i^{th}$ gene of parents: $\xi ^{k}_{Lbest}$ and $\xi ^{j}_{Lbest}$, respectively such that $k \ne j$. A new offspring is produced for each of *K* (which is equals to the total number of local blackholes) heuristic crossover. Equation (3) is applied to select best between new offspring ($\xi _{Off}$) and parent with lesser fitness ($\xi ^{j}_{Lbest}$). This allows to enhance the diversity of the local population with members of enriched fitness value.

3 $$\begin{aligned} \xi ^{j}_{Lbest}={\left\{ \begin{array}{ll} \xi _{Off} &{} \text {If (fitness}(\xi _{Off}) \ge {\hbox {fitness}} (\xi ^{j}_{Lbest})) \\ \xi ^{j}_{Lbest} &{} {\text {Otherwise}} \end{array}\right. } \end{aligned}$$

Thereafter, a best among the members of second phase population is nominated as global blackhole ($\xi ^{k}_{Gbest}$).

#### Position update

The position of stars is updated in accordance with $\xi ^{k}_{Lbest}$ and $\xi ^{k}_{Gbest}$ as depicted in Eq. (4), where $\xi _i^k(t)$ and $\xi _i^k(t+1)$ are the positions of $i$th star of $k$th sub population at time instances *t* and $t+1$, respectively. $r_1$ and $r_2$ are random numbers in the range (0, 1) while $\alpha _l$ and $\alpha _g$ are the attraction forces applied on $\xi _i^k(t)$ by $\xi ^{k}_{Lbest}$ and $\xi ^{k}_{Gbest}$, respectively. The inclusion of local best in position update procedure maintains the diversity of stars by gradually controlling the convergence speed and retains their exploratory behaviour.

4 $$\begin{aligned} Lf(t)= \alpha _1^k r_1\big (\xi ^{k}_{Lbest}(t)-\xi _i^k(t) \big )\\ Gf(t)= \alpha _gr_2\big (\xi ^{k}_{Gbest}(t) - \xi _i^k(t)\big ) \\ \xi _i^k(t+1)= \xi _i^k(t)+Lf(t) +Gf(t) \end{aligned}$$

The fitness value of all the updated stars is computed by applying Eq. (1). In case, if *k*th cluster locates a better solution than the existing one, the respective $\xi ^{k}_{Lbest}$ is replaced and $\xi ^{k}_{Gbest}$ is updated as per the admissibility. SB algorithm is inspired by the natural blackhole phenomenon, where a blackhole consumes everything that enters it including light. DPBHO algorithm works on the concept of a standard blackhole optimization algorithm, wherein none of the candidate solutions is allowed to return from an event horizon (*h*) area of a blackhole solution delineated by its radius ($\mathbb {R}_h$). The ratio between fitness value of a local blackhole ($f(\xi ^{k}_{Lbest})$) and fitness value of its sub-population ($\sum _{i=1}^{\mathbb {N}/K}{f(\xi ^{k}_{i})}$) computes the event horizon radius ($\mathbb {R}_h\big (\xi ^{k}_{Lbest}\big )$) of the respective blackhole as given in Eq. (5). Similarly, the event horizon radius of a global blackhole ($\mathbb {R}_h\big (\xi ^{k}_{Gbest}\big )$) is evaluated using Eq. (6), where $f(\xi ^{k}_{Gbest})$ is fitness value of global blackhole, $\sum _{k=1}^{K}\sum _{i=1}^{\mathbb {N}/K}{f(\xi ^{k}_{i})}$ is a fitness value of the entire population.

5 $$\begin{aligned}{} & {} \mathbb {R}_h\big (\xi ^{k}_{Lbest}\big )=\frac{f(\xi ^{k}_{Lbest})}{\sum _{i=1}^{\mathbb {N}/K}{f(\xi ^{k}_{i})}} \quad k \in [1, K] \end{aligned}$$

6 $$\begin{aligned}{} & {} \quad \mathbb {R}_h\big (\xi ^{k}_{Gbest}\big )=\frac{f(\xi ^{k}_{Gbest})}{\sum _{k=1}^{K}\sum _{i=1}^{\mathbb {N}/K}{f(\xi ^{k}_{i})}} \end{aligned}$$

The distance between both solutions is estimated by utilizing the arithmetic difference of their fitness values to confirm that a member solution has reached into the event horizon of the blackhole solution. The distance from local and global blackholes is calculated because each solution gets attracted to these two blackholes. Accordingly, the distance of *i*th star ($\xi ^{k}_{i}$) of *k*th sub-population from local blackhole ($\xi ^{k}_{Lbest}$) and global blackhole is computed in Eqs. (7) and (8), respectively.

7 $$\begin{aligned}{} & {} \mathbb {D}_{\xi ^{k}_{Lbest}}\big (\xi ^{k}_{i}\big )=f(\xi ^{k}_{Lbest}) - f(\xi ^{k}_{i}) \quad i \in [1, \mathbb {N}/K] \end{aligned}$$

8 $$\begin{aligned}{} & {} \quad \mathbb {D}_{\xi ^{k}_{Gbest}}\big (\xi ^{k}_{i}\big )=f(\xi ^{k}_{Gbest}) - f(\xi ^{k}_{i}) \quad i \in [1, 2K] \end{aligned}$$

If the distance between candidate solution $\xi ^{k}_{i}$ and local blackhole ($\xi ^{k}_{Lbest}$) is less than or equals to the event horizon radius of $\xi ^{k}_{i}$ i.e., $\mathbb {R}_h\big (\xi ^{k}_{Lbest}\big )$ then $\xi ^{k}_{i}$ gets collapse which is replaced by a new randomly generated solution to keep uniform number of solutions throughout the simulation. Following the same procedure, $\xi ^{k}_{i}$ gets collapse and replaced by a new random solution when it enters into the event horizon radius of the global blackhole $\mathbb {R}_h\big (\xi ^{k}_{Gbest}\big )$. The operational summary of DPBHO is given in Algorithm 1.
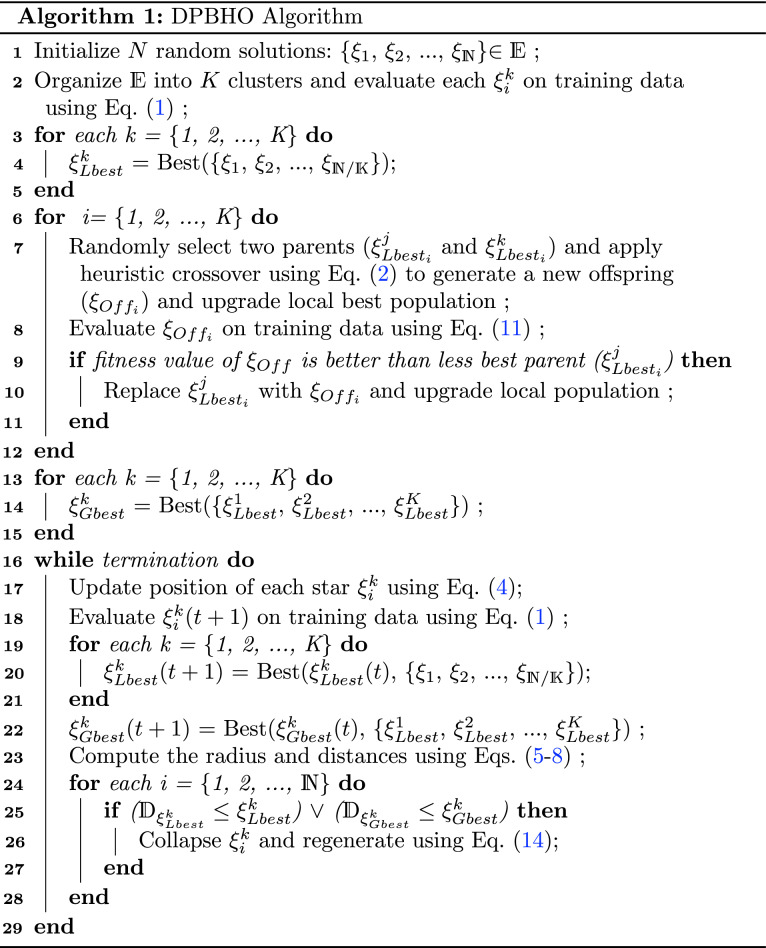


Step 1 initializes random solutions, and has complexity $\mathcal {O}(1)$. Step 2 evaluates the fitness of $\mathbb {N}$ solutions with $\mathcal {O}(\mathbb {N})$ complexity. Steps [3–5], steps [6–12], and steps [13–15] iterate *K* times and have equal time complexity of $\mathcal {O}(K)$. Assume steps [16–29] repeat for *t* intervals, wherein steps [19–21] have $\mathcal {O}(K)$ while steps [24–28] have $\mathcal {O}(\mathbb {N})$ complexities. Hence, the total time complexity for the DPBHO algorithm is $\mathcal {O}(\mathbb {N}Kt)$.

#### An illustration

Let there are 9 solutions (or stars) in the initial population ($E^1$) as shown in Table 9 which are grouped into 3 clusters during the first generation or epoch such that $Cluster_1^1$ (Table 10), $Cluster_2^1$ (Table 11), and $Cluster_3^1$ (Table 12). The fitness of each candidate solution is estimated using Eq. (11) and local best candidate is selected from each cluster. Likewise, $\xi _{Lbest_1}$, $\xi _{Lbest_2}$, and $\xi _{Lbest_3}$ constitute local best population (Table 13). The heuristic crossover operation is performed to improve the local best population using Eq. (2) and a global best candidate ($\xi _{Gbest}$) is chosen after fitness evaluation as depicted in Table 14. Further, the population is updated by computing event horizon radius for each cluster as well as a global radius of entire population as observed in Table 15. The distance of each candidate of the first generation population is estimated using Eqs. (7) and (8) to generate the next generation population as illustrated in Table 16, wherein the candidates $\xi _1$, $\xi _5$, $\xi _6$, and $\xi _8$, are updated.

**Table 9:** Initial generation population ($E^1$).

$\xi _1$:	− 0.94	− 0.66	− 0.84	− 0.22	− 0.126	− 0.99	− 0.13	− 0.15	− 0.71	0.06	− 0.03	− 0.60	0.20	− 0.07
$\xi _2$:	− 0.40	− 0.02	0.56	− 0.97	− 0.40	− 0.99	0.17	0.26	0.59	0.61	− 0.99	− 0.29	− 0.85	− 0.31
$\xi _3$:	− 0.49	− 0.41	− 0.58	− 0.70	− 0.59	0.17	− 0.94	− 0.64	− 0.08	− 0.02	− 0.88	0.18	0.09	0.23
$\xi _4$:	− 0.72	− 0.89	− 0.95	0.23	0.03	0.11	− 0.96	− 0.04	0.33	− 0.49	− 0.86	− 0.12	0.17	0.17
$\xi _5$:	0.37	0.56	− 0.51	− 0.89	− 0.39	0.89	0.37	− 0.54	0.58	− 0.92	0.77	0.04	0.03	0.24
$\xi _6$:	− 0.90	− 0.78	0.83	− 0.64	0.10	− 0.73	0.51	0.63	0.11	− 0.52	0.68	0.52	0.64	− 0.48
$\xi _7$:	− 0.81	− 0.52	− 0.76	0.63	− 0.80	− 0.19	0.36	0.59	0.61	0.19	− 0.45	− 0.85	− 0.96	0.26
$\xi _8$:	0.70	0.82	0.08	− 0.74	0.19	− 0.17	0.04	0.44	− 0.68	− 0.02	− 0.17	− 0.18	0.79	0.57
$\xi _9$:	− 0.47	− 0.41	0.51	0.23	− 0.39	0.09	0.38	0.54	− 0.08	− 0.12	0.37	0.54	0.67	− 0.24

**Table 10:** ($${Cluster^1_1}$$).

$\xi _1$:	− 0.94	− 0.66	− 0.84	− 0.22	− 0.126	− 0.99	− 0.13	− 0.15	− 0.71	0.06	− 0.03	− 0.60	0.20	− 0.07
$\xi _2$:	− 0.40	− 0.02	0.56	− 0.97	− 0.40	− 0.99	0.17	0.26	0.59	0.61	− 0.99	− 0.29	− 0.85	− 0.31
$\xi _3$:	− 0.49	− 0.41	− 0.58	− 0.70	− 0.59	0.17	− 0.94	− 0.64	− 0.08	− 0.02	− 0.88	0.18	0.09	0.23

**Table 11:** $${Cluster^1_2}$$.

$\xi _4$:	− 0.72	− 0.89	− 0.95	0.23	0.03	0.11	− 0.96	− 0.04	0.33	− 0.49	− 0.86	− 0.12	0.17	0.17
$\xi _5$:	0.37	0.56	− 0.51	− 0.89	− 0.39	0.89	0.37	− 0.54	0.58	− 0.92	0.77	0.04	0.03	0.24
$\xi _6$:	− 0.90	− 0.78	0.83	− 0.64	0.10	− 0.73	0.51	0.63	0.11	− 0.52	0.68	0.52	0.64	− 0.48

**Table 12:** $${Cluster^1_3}$$.

$\xi _7$:	− 0.81	− 0.52	− 0.76	0.63	− 0.80	− 0.19	0.36	0.59	0.61	0.19	− 0.45	− 0.85	− 0.96	0.26
$\xi _8$:	0.70	0.82	0.08	− 0.74	0.19	− 0.17	0.04	0.44	− 0.68	− 0.02	− 0.17	− 0.18	0.79	0.57
$\xi _9$:	− 0.47	− 0.41	0.51	0.23	− 0.39	0.09	0.38	0.54	− 0.08	− 0.12	0.37	0.54	0.67	− 0.24

**Table 13:** Local best population. ($\xi _{Lbest}^1$).

$\xi _{Lbest_1}$:	− 0.40	− 0.02	0.56	− 0.97	− 0.40	− 0.99	0.17	0.26	0.59	0.61	− 0.99	− 0.29	− 0.85	− 0.31
$\xi _{Lbest_2}$:	− 0.72	− 0.89	− 0.95	0.23	0.03	0.11	− 0.96	− 0.04	0.33	− 0.49	− 0.86	− 0.12	0.17	0.17
$\xi _{Lbest_3}$:	− 0.47	− 0.41	0.51	0.23	− 0.39	0.09	0.38	0.54	− 0.08	− 0.12	0.37	0.54	0.67	− 0.24

**Table 14:** Global best candidate ($\xi _{Gbest}^1$) after Heuristic Crossover.

$\xi _{Gbest}$:	− 0.80	0.32	− 0.70	− 0.85	− 0.40	− 0.79	0.69	0.21	0.40	0.41	− 0.52	− 0.27	− 0.75	− 0.61

**Table 15:** Event horizon computation.

Radius	Value
Local radius for $Cluster_1^1$	1.19350
Local radius for $Cluster_2^1$	1.75069
Local radius for $Cluster_3^1$	2.17435
Global radius	0.15525

**Table 16:** Second generation population ($E^2$).

$\xi _1$:	− 0.24	− 0.76	0.44	− 0.22	0.16	− 0.79	0.18	− 0.65	− 0.31	0.05	− 0.03	− 0.66	0.30	− 0.87
$\xi _2$:	− 0.40	− 0.02	0.56	− 0.97	− 0.40	− 0.99	0.17	0.26	0.59	0.61	− 0.99	− 0.29	− 0.85	− 0.31
$\xi _3$:	− 0.49	− 0.41	− 0.58	− 0.70	− 0.59	0.17	− 0.94	− 0.64	− 0.08	− 0.02	− 0.88	0.18	0.09	0.23
$\xi _4$:	− 0.72	− 0.89	− 0.95	0.23	0.03	0.11	− 0.96	− 0.04	0.33	− 0.49	− 0.86	− 0.12	0.17	0.17
$\xi _5$:	− 0.07	0.66	− 0.51	− 0.85	− 0.29	0.82	0.35	− 0.54	0.18	− 0.02	0.47	0.54	0.83	0.34
$\xi _6$:	0.92	0.73	− 0.88	− 0.64	0.16	− 0.23	0.71	− 0.03	0.15	0.52	− 0.68	− 0.82	0.24	− 0.62
$\xi _7$:	− 0.81	− 0.52	− 0.76	0.63	− 0.80	− 0.19	0.36	0.59	0.61	0.19	− 0.45	− 0.85	− 0.96	0.26
$\xi _8$:	− 0.75	0.02	− 0.58	0.44	0.18	− 0.12	0.04	0.84	− 0.48	− 0.02	− 0.67	− 0.18	− 0.79	− 0.92
$\xi _9$:	− 0.47	− 0.41	0.51	0.23	− 0.39	0.09	0.38	0.54	− 0.08	− 0.12	0.37	0.54	0.67	− 0.24

### Cloud workload resource usage analysis

The cloud workload analysis comprises of two steps: data preparation and multi-layered feed-forward neural network (MFNN) optimization using DPBHO algorithm as described in detail in the following subsections.

#### Data preparation

MFNN derives intial information for data preparation from Historical Resource Usage database of different clusters {$CS_1$, $CS_2$, …, $CS_n$} which is updated periodically with live resource usage information as portrayed in block CW-RUA of Fig. 7. Let the received historical resource usage information: {$d_1$, $d_2$ , …, $d_z$}: $\in \varpi ^{In}$ is aggregated with respect to a specific time-interval (for example, 1 min, 5 min, 10 min, 60 min and so on). The aggregated values have high variance which are rescaled within the range [0.001, 0.999] by applying Eq. (9), where $\varpi ^{In}_{min}$ and $\varpi ^{In}_{max}$ are the minimum and maximum values of the input data set, respectively. The normalized vector is denoted as $\hat{\varpi ^{In}}$, which is a set of all normalized input data values as $\hat{\varpi ^{In}}$.

9 $$\begin{aligned} \hat{\varpi ^{In}}= 0.001 +\frac{ d_i- \varpi ^{In}_{min}}{\varpi ^{In}_{max}-\varpi ^{In}_{min}}\times (0.999) \end{aligned}$$

These normalized values (in single dimension) are organized into two dimensional input and output matrices denoted as $\varpi ^{In}$ and $\varpi ^{Out}$, respectively as stated in Eq. (10):

10 $$\begin{aligned} \varpi ^{In}= \left[ {\begin{array}{cccc} \varpi _1 &{} \varpi _2 &{} .... &{} \varpi _z\\ \varpi _2 &{} \varpi _3 &{} .... &{} \varpi _{z+1} \\ . &{} . &{} .... &{} . \\ \varpi _m &{} \varpi _{m+1} &{} .... &{} \varpi _{z+m-1} \\ \end{array} } \right] \varpi ^{Out}= \left[ {\begin{array}{c} \varpi _{z+1} \\ \varpi _{z+2} \\ . \\ \varpi _{z+m} \end{array} } \right] \end{aligned}$$

#### MFNN optimization

The prepared data values $\varpi ^{In}$ are divided into three groups: training (60%), testing (20%), and validation (20%) data, where training data is used to optimize the predictor while testing data is used for evaluating the prediction accuracy over unseen data. During training, MFNN extracts intuitive patterns from actual workload ($\varpi ^{In}$) and analyzes *z* previous resource usage values to predict the $(z+1)$th instance of workload in each pass. In the course of training and testing period, the performance and accuracy of the proposed model is evaluated by estimating the *Mean Squared Error* ($\varpi ^{MSE}$) score as fitness function) using Eq. (11); where $\varpi ^{AO}$ and $\varpi ^{PO}$ are actual and predicted output, respectively^25^. Further, validation data is applied to confirm the accuracy of the proposed prediction model, wherein *Mean absolute error* ($\varpi ^{MAE}$) stated in Eq. (12) is used as a fitness function because it is an easily interpretable and well established metric to evaluate regression models.

11 $$\begin{aligned} \varpi ^{MSE}= & {} \frac{1}{m}\sum _{i=1}^{m}(\varpi ^{AO}_{i}-\varpi ^{PO}_{i})^2 \end{aligned}$$

12 $$\begin{aligned} \varpi ^{MAE}= & {} \sum _{i=1}^{m}{\frac{\Vert \varpi ^{AO}_{i}-\varpi ^{PO}_{i}\Vert }{m}} \end{aligned}$$

In the proposed approach, MFNN represents a mapping *p*-$q_1$-$q_2$-$q_3$-*r*, wherein *p*, $q_1$, $q_2$, $q_3$ and *r* are the numbers of neurons in input, hidden$\#1$, hidden$\#2$, hidden$\#3$, and output layer, respectively. Since the output layer has only one neuron, the value of *r* is constantly 1. The activation function used to update a neuron is stated in Eq. (13), where a linear function ($(\varpi )$) is applied to input layer neurons and sigmoid function ($\frac{1}{1+e^{-\varpi }}$) for the rest of the neural layers.

13 $$\begin{aligned} f(\varpi )={\left\{ \begin{array}{ll} \varpi &{} \text {If (Input layer)} \\ \frac{1}{1+e^{-\varpi }} &{} {\text {otherwise.}} \end{array}\right. } \end{aligned}$$

The training begins with randomly generated $\mathbb {N}$ networks of real-numbered vectors denoted as {$\xi _1$, $\xi _2$, …, $\xi _\mathbb {N}$}$\in \mathbb {E}$, wherein each vector ($\xi _i: 1 \le i \le \mathbb {N}$) has size $L=$(($p+1$)$\times q_1+ q_1 \times q_2 +q_2 \times q_3 + q_3 \times r$). The number of neurons in input layer become $p+1$ by reason of consideration of one additional bias neuron. The synaptic or neural weights ($W^*_{ij}$) are generated randomly with uniform distribution as shown in Eq. (14), where $lb_j =-1$ and $ub_j =1$ are the lower and upper bounds, respectively and *r* is a random number in the range [0, 1].

14 $$\begin{aligned} W^*_{ij} = lb_j + r \times (ub_j - lb_j) \end{aligned}$$

MFNN is optimized periodically using DPBHO by considering each network vector ($\xi _i: 1 \le i \le \mathbb {N}$) as a star, where Eq. (11) is applied as a fitness function and the candidate having least fitness value is nominated as a best candidate both in local and global population optimization phase.

### Secure and sustainable VM placement

Let $\omega$ represents a mapping between VMs and servers such that $\omega _{kji}=1$, if server $S_i$ hosts $V_j$ of $k^{th}$ user, else it is 0 as stated in Eq. (15).

15 $$\begin{aligned} \omega _{kji}={\left\{ \begin{array}{ll} 1 &{} \text {If (VM }V_j \text { of } k\text {th user is hosted on server }S_i) \\ 0 &{} {\text {Otherwise.}} \end{array}\right. } \end{aligned}$$

The essential set of constraints that must be satisfied concurrently have been formulated in Eq. (16):

16 $$\begin{aligned} \left. \begin{array}{ll} C_1: \quad \sum _{k \in M}\sum _{j \in Q}\sum _{i \in P}{\omega _{kji}}=1\\ C_2: \quad \sum _{k \in M} \sum _{j \in Q}\sum _{i \in P}{V_j^{C}} \times \omega _{kji} \le S_i^{C^{*}}\\ C_3: \quad \sum _{k \in M}\sum _{j \in Q}\sum _{i \in P}{V_j^{M}} \times \omega _{kji} \le S_i^{M^{*}}\\ C_4: \quad \sum _{k \in BW}\sum _{j \in Q}\sum _{i \in P}{V_j^{BW}} \times \omega _{kji} \le S_i^{BW^{*}}\\ C_5: \quad \sum _{k\in M}{R}_k \le \sum _{i \in P}S_i^{\mathbb {R^{*}}} \quad R^{*} \in \{C^{*}, M^{*}, BW^{*}\} \\ C_6: \quad r_k \times {R}_k \le V_j^{R^*} \quad \forall _k \in [1, M], j \in [1, Q] \end{array} \right\} \end{aligned}$$

where $C_1$ implies $j^{th}$ VM of *k*th user must be deployed only on one server. The constraints $C_2$, $C_3$, $C_4$ state that $j$th VM’s CPU ($V_j^{C}$), memory ($V_j^{M}$), and bandwidth ($V_j^{BW}$) requirement must not exceed available resource capacity of $i$th server ($S_i^{C^{*}}$, $S_i^{M^{*}}$, $S_i^{BW^{*}}$). $C_5$ specifies that aggregate of the resource capacity request of all the users must not exceed total available resources capacity of the servers altogether. $C_6$ states that required resource capacity (${R}_k$) of request $r_k$ must not exceed total available resources capacity ($R^{*} \in \{C^{*}, M^{*}, BW^{*}\}$) of VM $V_j$.

The considered load management problem in CDC entangled with multiple constraints seeks to provide a secure and energy-efficient VM placement. Accordingly, a multi-objective function for allocating VMs is stated in Eq. (17):

17 $$\begin{aligned} Minimize:\quad f_{\Xi _{{CDC}}}\big (\omega _{kji} \big ), f_{PW_{{CDC}}}\big (\omega _{kji} \big ),\\ f_{PUE_{CDC}}\big (\omega _{kji} \big ), f_{{CFR_{{CDC}}}}\big (\omega _{kji} \big ), \\ Maximize: \quad f_{RU_{{CDC}}}\big (\omega _{kji} \big ) \quad {s.t. \quad \{C_1-C_6\} } \end{aligned}$$

Likewise, the following five distinct models associated to each objective are designed and utilized to establish a secure and sustainable VM placement scheme for CDC.

#### Security modeling

The sharing of servers among different users is minimized by reducing the allocation of VMs of different users on a common physical server to resist the probability of security attack via co-resident malicious VMs. The probability of occurrence of security attacks is represented as $\Xi$. Let $\beta _{ki}$ specifies a mapping between user $U_k$ and server $S_i$, whereif a server hosts VMs of more than one user then $\beta _{ki}=1$, otherwise it is 0. The total number of users having their VMs located on server $S_i$ are obtained by computing $\sum _{k=1}^{M}{\beta _{ki}}$. The number of shared server percentile is referred as $\Xi$ which is be computed over time-interval {$t_1$, $t_2$} by using Eq. (18). In contrast to existing secure VM allocation scheme^26^, the proposed security model is capable of reducing co-residential vulnerability threats without any prior information of malicious user and VM.

18 $$\begin{aligned} \Xi _{CDC}= \int \limits _{\begin{array}{c} t_1\\ \mathcal {} \end{array}}^{t_2}\bigg (\frac{\sum _{i=1}^{P}\sum _{k=1}^{M}{\beta _{ki}}}{|S|} \bigg )dt \times 100 ; \quad \forall \sum _{k=1}^{M}{\beta _{ki} > 1} \end{aligned}$$

#### Server resource utilization modeling

Let $S_i^{C}$, $S_i^{Mem}$ and $S_i^{BW}$ be the CPU, memory, and bandwidth capacity, respectively for $i^{th}$ server and $V_j^{C}$, $V_j^{Mem}$ and $V_j^{RAM}$ represents CPU, memory, and bandwidth utilization, respectively for $j$th VM. When $S_i$ is active, $\Upsilon _i=1$, otherwise it is 0. CPU, memory and bandwidth utilization of a server can be estimated by applying Eqs. (19)–(21).

19 $$\begin{aligned} RU_i^{C}=\frac{\sum _{j=1}^{Q}{\omega _{ji} \times V_j^{C}}}{S_i^{C}} \end{aligned}$$

20 $$\begin{aligned} {RU_i}^{Mem}=\frac{\sum _{j=1}^{Q}{\omega _{ji} \times V_j^{Mem}}}{S_i^{Mem}} \end{aligned}$$

21 $$\begin{aligned} {RU_i}^{BW}=\frac{\sum _{j=1}^{Q}{\omega _{ji} \times V_j^{BW}}}{S_i^{BW}} \end{aligned}$$

Equation (22) calculates resources utilization of server ($RU_{S_i}^{\mathbb {R}}: \{C, Mem, BW\} \in \mathbb {R}$) and complete resource utilization of data centre ($RU_{CDC}$) is determined by applying Eq. (23) where, *N* is the number of resources observed.

22 $$\begin{aligned} RU_{S_i}^{\mathbb {R}}= & {} {RU_{S_i}^{C} +RU_{S_i}^{M} + RU_{S_i}^{BW}} \end{aligned}$$

23 $$\begin{aligned} RU_{CDC}= & {} \frac{\sum _{i=1}^{P}RU_{S_i}^{\mathbb {R}}}{|N|\times \sum _{i=1}^{P}{\Upsilon _i}} \end{aligned}$$

#### Server power consumption modeling

Consider all the servers based on inbuilt Dynamic Voltage Frequency Scaling (DVFS) energy saving technique^27^ which defines two states of CPU: *inactive* and *active* state. In active state, CPU works in least operational mode with reduced clock cycle and some internal components of CPU are set inactive. On the other hand, in active state, power consumption depends on the CPU utilization rate and processing application. Therefore, power consumption for a server can be formulated as $PW_{S_i}$ for $i^{th}$ server and total power consumption $PW_{CDC}$ for time-interval {$t_1$, $t_2$} as given in Eqs. (24) and (25), respectively, where $RU_{S_i} \in$ [0, 1] is resource utilization of server ($S_i$).

24 $$\begin{aligned} PW_{S_i}= & {} ([{PW_{S_i}}^{max} - {PW_{S_i}}^{min}] \times RU_{{S_i}} + {PW_{S_i}}^{idle}) \end{aligned}$$

25 $$\begin{aligned} PW_{CDC}= & {} \sum _{i=1}^{P} {PW_{S_i}} \end{aligned}$$

#### Power usage effectiveness

This is a very significant metric for measuring power efficiency of CDC. It is expressed as ratio of the total power supply ($PW^{total}_{S_i}$) of a server ($S_i$) to run its processing equipments and other overheads like cooling and support systems and effective power utilized ($PW^{utilized}_{S_j}$) by it. Equations (26) and (27) calculate the power usage effectiveness of a server $S_i$ and CDC, respectively.

26 $$\begin{aligned} PUE(S_{i})= & {} \frac{PW^{total}_{S_i}}{PW^{utilized}_{S_j}}=\frac{PW^{others}_{S_j} + PW^{utilized}_{S_j}}{PW^{utilized}_{S_j}} \end{aligned}$$

27 $$\begin{aligned} PUE_{CDC}= & {} \sum _{i=1}^{P} {PUE(S_{i})} \end{aligned}$$

### Carbon foot-print rate

The carbon emission intensity varies in accordance with source of electricity generation. Here, the variables $\mathbb {S}$, $\mathbb {W}$, and $\mathbb {N}$ refer to carbon intensity of the energy sources: solar, wind and non-renewable energy sources, respectively. The carbon intensity is measured in Tons per Mega Watt hour (Tons/MWh) electricity used. The emission of carbon dioxide in the environment directly depends on the carbon intensity represented as $CFR(V_j)$ and computed by applying in Eq. (28)^4^:

28 $$\begin{aligned} CFR(V_{j})= \sum _{x \in \{\mathbb {S}, \mathbb {W}, \mathbb {N}\}}\big (E_{RU, x} + E_{others, x}\big ) \times RU_x^{E} \end{aligned}$$

### VM management

**Figure 9:**
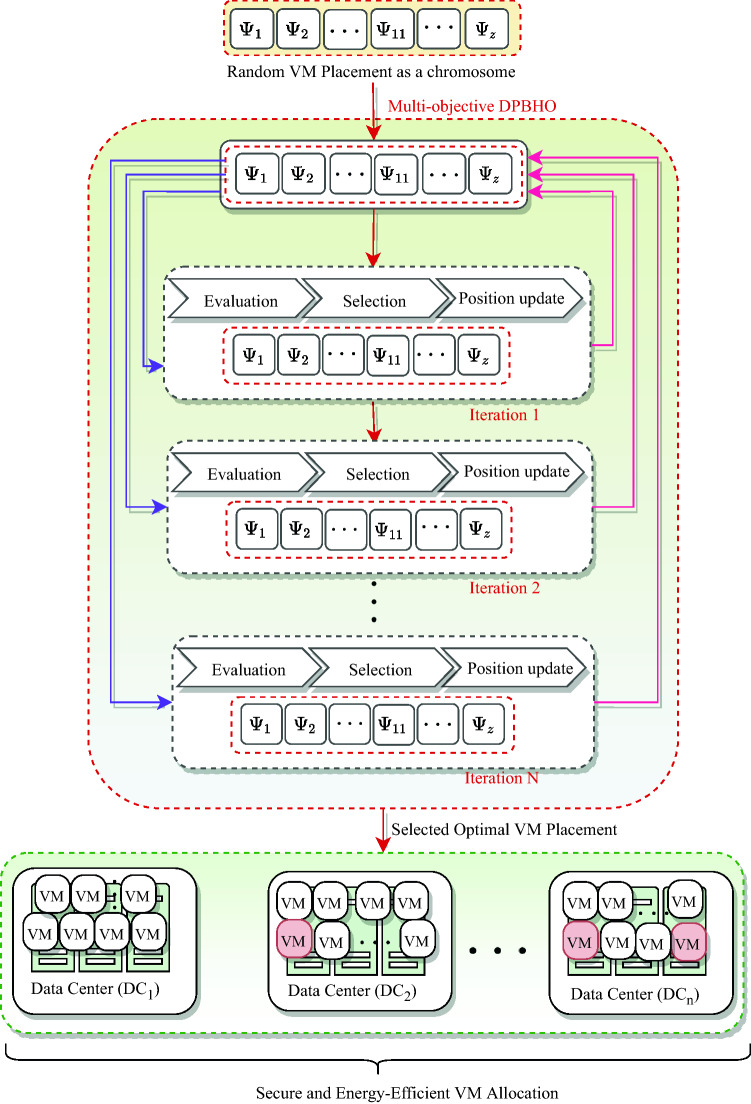
Multi-objective DPBHO based VM placement.

The VMs are allocated by utilizing Multi-objective DPBHO (i.e., M-DPBHO) which is an integration of proposed DPBHO algorithm and pareto-optimal selection procedure of Non-dominated Sorting based Genetic Algorithm (NSGA-II)^28^. M-DPBHO comprises of steps: (i) *initialization*, (ii) *evaluation*, (iii) *selection*, and (iv) *position update*. As illustrated in Fig. 9, *X* VM allocations represented as stars/solutions: {$\Psi ^g_1$, $\Psi ^g_2$, …, $\Psi ^g_X$}$\in \Psi$ are randomly *initialized*, where *g* is the number of generation. These stars are *evaluated* using a fitness function $\eta (\Psi ^g)=$ [$f(\Psi ^g)_{\Xi _{{CDC}}}$, $f(\Psi ^g)_{PW_{{CDC}}}$, $f(\Psi ^g)_{PUE_{{CDC}}}$, $f(\Psi ^g)_{CFR_{{CDC}}}$, $f(\Psi ^g)_{RU_{{CDC}}}$] associated with security [Eq. (18)], power consumption [Eq. (25)], power usage effectiveness [Eq. (27)], carbon-foot rate [Eq. (28)], and resource utilization [Eq. (23)], respectively.

The population of stars is distributed into *K* sub-populations and local best blackholes ($\Psi ^k_{Lbest}$) are selected by estimating the fitness value using pareto-optimal selection procedure of NSGA-II. Thereafter, a second phase population is generated with the help of heuristic crossover [using Eq. (2)]. Similar to the local phase, a global best solution ($\Psi ^k_{Gbest}$) is observed from the second phase population using pareto-optimal procedure.

Therefore, to *select* the best VMP solution, a pareto-front selection procedure of NSGA-II is invoked that concedes all the objectives non-dominantly. A solution ($\Psi _i$) dominates other solution ($\Psi _j$), if its fitness value is better than that of $\Psi _j$ on atleast one objective and same or better on other objectives. The position update step of DPBHO [including Eq. (4)] along with Eqs. (5) and (6) for computing event horizon radius of local and global blackholes, respectively while Eqs. (7) and (8) are used to determine distance of a candidate solution from a local and global blackhole, respectively) is invoked to regenerate or update the existing population. Let a user job request ($\lambda$) is distributed into sub-units or tasks such as {$\tau _1$, $\tau _2$, …, $\tau _z$}$\in \lambda$. Eq. (29) is employed to select an appropriate VM for user application execution,

29 $$\begin{aligned} VM^{type}_{selected} = {\left\{ \begin{array}{ll} V_{S}, &{} {(\tau ^{\mathbb {R}}_{i} \le V_{S}^{\mathbb {R}} )} \\ V_{M}, &{} {( V_{S}^{\mathbb {R}}< \tau ^\mathbb {R}_{i} \le V_{M}^{\mathbb {R}})} \\ V_{L}, &{} {(V_{M}^{\mathbb {R}} < \tau ^\mathbb {R}_{i} \le V_{L}^{\mathbb {R}})} \\ V_{XL}, &{} \text {otherwise} \end{array}\right. } \end{aligned}$$

where $V_{S}^{\mathbb {R}}$, $V_{M}^{\mathbb {R}}$, $V_{L}^{\mathbb {R}}$ and $V_{XL}^{\mathbb {R}}$ represents small, medium, large and extra-large types of VM respectively, having capacity of resources $\mathbb {R} \in \{CPU, memory\}$ depending on their particular type, and $\tau ^\mathbb {R}_{i}$ represents resource utilization of $i$th task. If the maximum resource requirement of a task from $i$th task is lesser or equals to the resource capacity of $V_{S}$, then small type of VM is assigned to the task.

### SaS-LM: operational design and complexity

Algorithm 2 elucidates a concise operational design of SaS-LM. Step 1 initializes list of VMs ($List_{\mathbb {V}}$), list of servers ($List_{\mathbb {S}}$), list of users ($List_{\mathbb {U}}$), and iteration counter (*g*) with *O*(1) complexity. Step 2 optimizes MFNN based predictor for resource usage analysis by invoking Algorithm 1 having *O*(*XKt*) complexity for *t* time-intervals. The steps 3–31 repeat for $\Delta t$, wherein any resource contention is detected and mitigated with the help of steps 4–9 with *O*(*P*) complexity. Step 10 receives live requests of users has *O*(1) complexity. Steps 11–13 select suitable VMs for requests execution with *O*(*Q*) complexity. *X* VM allocations are randomly initialized in step 14 with *O*(*X*) complexity.
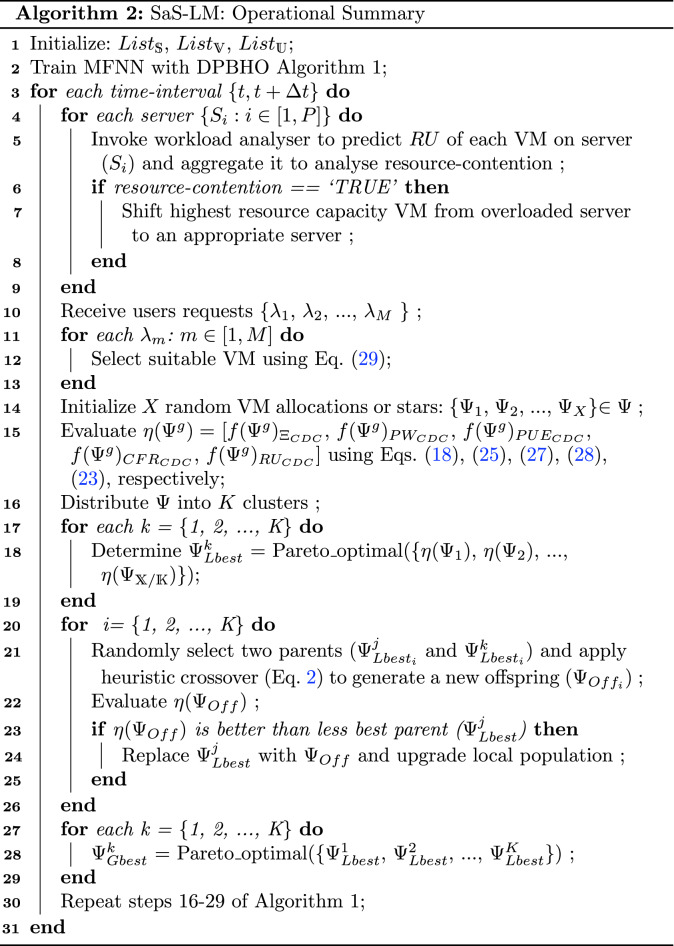


The cost values associated to five objectives is computed in step 15, where complexity is *O*(*X*) and step 16 distributes *X* VM allocations into *K* with *O*(1) complexity. The best VM allocation candidate is selected in steps 17–19 by invoking Pareto-optimal function have $O(X^2)$ complexity. The local population of VM allocations is upgraded using heuristic crossover in steps 20-26, consume *O*(*K*) complexity. Further, the cost values of second phase population (as mentioned in DPBHO Algorithm) is evaluated and global best candidate is selected in steps 27–29 with $O(K^2)$ complexity. Step 30 invokes set of instructions 16–29 of Algorithm 1, have *O*(*KX*) complexity. The total complexity of SaS-LM becomes $O(X^2K^2PQt)$.

#### Implementation

**Figure 10:**
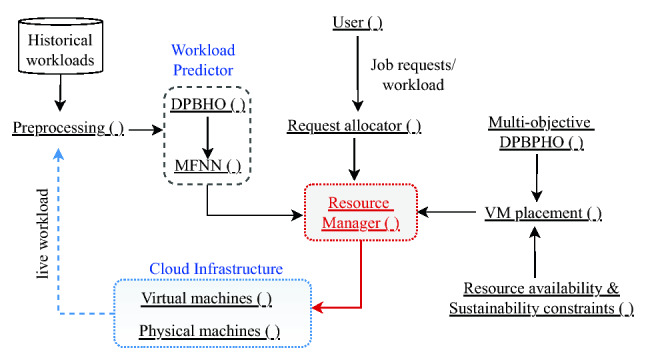
Design and operational flow.

Figure 10 portrays a design and operational flow of the proposed model. Specifically, SaS-LM model is configured with the cooperative interaction of the distinguished modules discussed as follows:

- *Preprocessing* (): The relevant numerical values of historical and live workloads are extracted and normalized to prepare input values for training of workload predictor.
- *Workload Predictor*: This module is employed to estimate future resource usage on different servers with the help of multi-resource feed-forward neural network *MFNN* () module. This neural network is trained (offline) periodically to precisely estimate the approaching job requests in real-time to provide prior information to the *Resource Manager* () about the required amount of resources and alleviate any delay in job processing.
- *DPBHO* (): This module implements Algorithm 1 for optimization of MFNN based predictor during training or learning process.
- *User *(): User assigns job requests to *Requests allocator* () module at regular intervals for execution on different VMs. It also specifies deadline, cost, security, and resource availability constraints in Service Level Agreement (SLA).
- *Virtual machines* (): As per the demand of the users, varying types of VM instances with specific configuration such as CPU, storage, bandwidth, operational status etc. are configured and allocated to servers.
- *Physical machines* (): The varying types of servers configuration is defined by specifying their CPU, storage, bandwidth, operational status etc.
- *Resource availability and Sustainability constraints* (): The security and sustainability constraints depict the computational models mentioned in Section “Secure and sustainable VM placement” which are considered non-dominantly to decide the most admissible allocation of VMs.
- *Multi-objective DPBHO* (): This module appoints the VM placement strategy mentioned in Section “VM Management” to explore and exploit the population of random VM allocations and select the best VM placement.
- *Resource Manager* (): This module receives essential information from different modules including Resource allocator (), Multi-objective DPBHO based VM placement, predicted resource capacity from MFNN (). Accordingly, it decides the allocation of available physical machines and manage the resources adaptively.

## Background and discussion

The background study deals with discussion of several approaches proposed thus far for cloud resource provisioning using meta-heuristic approaches^29^ and machine learning algorithms for cloud workload analysis^30^. An online prediction based multi-objective load-balancing (OP-MLB) framework is proposed in^18^ for energy-efficient data centres. The forthcoming load on VMs is estimated using an Auto Adaptive Differential Evolutionary (AADE) trained neural network-based prediction system to determine the future resource utilization of the servers proactively. Also, it detected an overload condition on each server and tackled it by migrating VMs of highest resource capacity from overloaded server to an energy-efficient server machine. The VM placement and migration are executed using a non-dominated sorting with genetic algorithm based multi-objective algorithm for minimization of power consumption. A distributive UPS topology at server-level and rack-level based framework for cloud resource management is proposed in^14^. This framework established VM placement, appropriate time of battery charging and discharging, and selected a battery that minimizes the peak demands and monthly electricity bill. The VM requests are scheduled by developing a Slack and Battery Aware (SBA) placement based on power state of the servers, resource utilization, and the amount of energy stored in server batteries. It helped to reduce the number of active servers and maximize the accessible stored energy to be utilized during peak demands.

Dabbagh et al.^21^ presented an integrated energy-efficient VM placement and migration framework for cloud data centre. It applied a Wiener filter with safety margin (WP-SM) based prediction for estimation of the number of VM requests and the future resource requirement. These predicted values are used to allow only the required number of physical machines in active state and helps in achieving a substantial energy saving and resource utilization. Kaur et al.^4^ have presented a Boruta algorithm driven multi-objective optimization scheme based job scheduling (BM-JS) along with energy-efficient VM placement for sustainable cloud environment. Specifically, they have classified upcoming workload using Boruta algorithm and sensitive hashing-based support vector machines approach followed by Greedy scheme based VM placement to reduce carbon footprint and energy consumption. A secure and multi-objective VM placement (SVMP) framework is proposed in^20^, where an integrated version of whale optimization algorithm and non-dominated sorting based genetic algorithm is implemented to attain multiple objectives concurrently. Marahatta et al.^17^ have proposed a failure management aware cloud resource distribution approach named Prediction based Energy-aware Fault-tolerant Scheduling scheme (PEFS). Specifically, a deep neural network based failure predictor is utilized to differentiate between failure prone and non-failure prone tasks. Three replicas are executed for failure-prone tasks on separate servers to prevent redundant execution on the same server while non-failure tasks execute normally. Nguyen et al.^15^ addressed the VM consolidation problem by adopting multiple usage prediction by applying multiple linear regression to estimate the relationship between the input variables and the output for energy efficient data centres. This work estimated overloaded host detection with multiple usage prediction (OHD-MUP) and underloaded host detection with multiple usage prediction (UHD-MUP) and balanced load by migrating selected VMs from overloaded servers to energy-efficient server.

A metaheuristic technique-based Fuzzy C-means clustering (MTFC) mechanism is proposed in^31^ to locate most promising clusters according to the users’ Quality-of-Service (QoS) requirement. Further, a gray wolf optimization is applied to make an appropriate scaling decision for cloud resource provisioning. Tarahomi et al.^32^ have proposed a micro-genetic approach (MGA) to present power-efficient resource distribution of physical resources for sustainable cloud services. The micro-genetic algorithm helps to select suitable destinations for VMs amongst physical hosts. Likely, a resource elasticity management issue is resolved in^33^ by proposing an elastic controller based on colored Petri Nets (EC-CPN) that assists in automatic handling of over-/under-provisioning of resources. A co-location resistant VM placement method, “Previously Co-Located Users First” (PCUF) is presented in^16^ where VMs are placed and co-located according to their user identities of previous allocation in order to reduce the co-residency attacks. A Link Based Virtual Resource Management (LVRM) algorithm is proposed in^22^ which employed a mapping of virtual links and nodes for reduction of their impact on request execution time to minimize the number of active servers. It assigned a highest priority to the virtual link having maximum network bandwidth to minimize the execution time of request. Also, it assigned multiple VMs to a single server by applying Dijkstra algorithm for selection of the substrate path between two servers so as to enhance the request execution rate. To meet dynamic demands of the future applications, an energy-efficient resource provisioning framework is developed in^19^. This framework addressed the challenges including resource wastage, degradation of performance and QoS by comparing the application’s predicted resource requirement with resource capacity of VMs and consolidating entire load on the minimum number of servers. An online multi-resource feed-forward neural network (OM-FNN) is developed and optimized with Tri-adaptive Differential Evolutionary (TaDE) algorithm to forecast the multiple resource demands and predicted VMs are placed on energy-efficient servers. This integrated approach optimized resource utilization and energy consumption.

Majority of the existing works have investigated sustainability of CDCs with respect to energy consumption only and few others have studied resource utilization while ignoring carbon emission, power usage efficiency, which are essential credentials to be considered during sustainable resource management. Further, none of the prior works have considered security along with sustainability during VM consolidation. In the light of the existing approaches, the proposed SaS-LM model addresses multiple objectives associated to sustainability of CDCs as well as considers security of users’ applications under processing in real-time. The proposed DPBHO algorithm training based workload analyser learns resource usage patterns and characteristics with precise accuracy to allow enhanced utilization of servers, PUE, and reduced carbon emission. Also, multi-objective DPBHO based VM management consolidates VMs on most efficient servers which caters multiple objectives for enhanced sustainability of CDCs with usage of green power supply while meeting QoS constraints simultaneously. Table 17 compares the SaS-LM model with state-of-the-art approaches thoroughly.

**Table 17:** Comparison of SaS-LM model with state-of-the-art approaches.

Model	Approach		Objectives					Evaluation		Remarks
	WP$^*$	LM$^*$	$\Xi$	*RU*	*PW*	*PUE*	*CFR*	Dataset	Tool	
OP-MLB^18^	NN	$\checkmark$	$\times$	$\checkmark$	$\checkmark$	$\times$	$\times$	GCD, PLB, BB	Python	CPU temperature, CFP, & security were ignored
SBA^14^	$\times$	$\checkmark$	$\times$	$\checkmark$	$\checkmark$	$\times$	$\times$	GCD	CloudSim	Battery-aware approach only, PUE, CFP ignored
WPSM^21^	Wiener Filter	$\checkmark$	$\times$	$\checkmark$	$\checkmark$	$\times$	$\times$	GCD	CloudSim	Adoption of weak approach for overload prediction, security lacking
BM-JS^4^	$\times$	$\checkmark$	$\times$	$\checkmark$	$\checkmark$	$\checkmark$	$\checkmark$	GCD	CloudSim	Task elasticity is exploited, but overload handling is ignored
SVMP^20^	$\times$	$\checkmark$	$\checkmark$	$\checkmark$	$\checkmark$	$\times$	$\times$	GCD	Python	Resource contention and overload handling are ignored
PEFS^17^	DNN	$\checkmark$	$\times$	$\checkmark$	$\checkmark$	$\times$	$\times$	GCD	Python	Security and over-/under-load handling are ignored
MUP^15^	LR	$\checkmark$	$\times$	$\checkmark$	$\checkmark$	$\times$	$\times$	GCD, PLB	Java	Security and system sustainability perspectives are missing
MTFC^31^	$\times$	$\checkmark$	$\times$	$\checkmark$	$\times$	$\times$	$\times$	GCD, internet	CloudSim	Task elasticity is exploited, overload handling and other aspects ignored
MGA^32^	$\times$	$\checkmark$	$\times$	$\times$	$\checkmark$	$\times$	$\times$	PLB	CloudSim	Power consumption minimized but ignored resource wastage
EC-CPN^33^	$\times$	$\checkmark$	$\times$	$\checkmark$	$\times$	$\times$	$\times$	GCD, Yahoo, Wiki.	CPN Tools + Cloudsim	Task elasticity is considered, over-/under-load handling concepts are ignored
PCUF^16^	$\times$	$\checkmark$	$\checkmark$	$\checkmark$	$\times$	$\times$	$\times$	Azure traces	CloudSim	May suffer from security breaches not based on previous co-locations
LVRM^22^	$\times$	$\checkmark$	$\times$	$\checkmark$	$\times$	$\times$	$\times$	Artificial traces	CVI-Sim (java)	Bandwidth usage of a task is given higher priority over computing
OM-FNN^19^	NN	$\checkmark$	$\times$	$\checkmark$	$\checkmark$	$\times$	$\times$	GCD	Python	Underload handling provisions are ignored
SaS-LM	MFNN+ DPBHO	$\checkmark$	$\checkmark$	$\checkmark$	$\checkmark$	$\checkmark$	$\checkmark$	GCD	Python	Provides secure & sustainable LM where trust & reliability can be included to improve security

WP$^*$: Workload prediction, LM$^*$: Load management, NN: Neural network, DNN: Deep neural network, LR: Linear Regression, GCD: Google Cluster Dataset, PLB: Planet Lab VM traces, BB: Bitbrains VM traces.

## Conclusion and future work

A novel SaS-LM model is proposed to provide a pareto-optimal solution for secure and sustainable workload management in the green cloud environment. The model incorporates a newly developed DPBHO evolutionary optimization algorithm for neural network-based resource usage estimation. Further, Multi-objective DPBHO-based real-time VM placement and management are presented to serve the perspectives of both the cloud user and service provider, concurrently. There is a substantial reduction in security attacks, carbon emission, and power consumption with an improvement in resource utilization and PUE. The achieved results show superiority of SaS-LM model compared to the existing state-of-the-art approaches. Also, a trade-off is observed revealing that sustainability improves at the cost of security and vice-versa. In the future, the proposed model can be extended by prioritizing the objectives as per the dynamic requirement, adding objectives like trust and reliability-based VM allocation scheme.

## Data Availability

The dataset used and/or analysed during the current study available from the corresponding author on reasonable request.
